# Artificial Intelligence in Clinical Medicine: Challenges Across Diagnostic Imaging, Clinical Decision Support, Surgery, Pathology, and Drug Discovery

**DOI:** 10.3390/clinpract15090169

**Published:** 2025-09-16

**Authors:** Eren Ogut

**Affiliations:** Department of Anatomy, Faculty of Medicine, Istanbul Medeniyet University, Istanbul 34700, Türkiye; erenogut@yahoo.com.tr or eren.ogut@medeniyet.edu.tr; Tel.: +90-2162803333

**Keywords:** artificial intelligence, machine learning, deep learning, diagnostic imaging, clinical decision support, surgery, pathology, drug discovery

## Abstract

**Aims/Background:** The growing integration of artificial intelligence (AI) into clinical medicine has opened new possibilities for enhancing diagnostic accuracy, therapeutic decision-making, and biomedical innovation across several domains. This review is aimed to evaluate the clinical applications of AI across five key domains of medicine: diagnostic imaging, clinical decision support systems (CDSS), surgery, pathology, and drug discovery, highlighting achievements, limitations, and future directions. **Methods:** A comprehensive PubMed search was performed without language or publication date restrictions, combining Medical Subject Headings (MeSH) and free-text keywords for AI with domain-specific terms. The search yielded 2047 records, of which 243 duplicates were removed, leaving 1804 unique studies. After screening titles and abstracts, 1482 records were excluded due to irrelevance, preclinical scope, or lack of patient-level outcomes. Full-text review of 322 articles led to the exclusion of 172 studies (no clinical validation or outcomes, *n* = 64; methodological studies, *n* = 43; preclinical and in vitro-only, *n* = 39; conference abstracts without peer-reviewed full text, *n* = 26). Ultimately, 150 studies met inclusion criteria and were analyzed qualitatively. Data extraction focused on study context, AI technique, dataset characteristics, comparator benchmarks, and reported outcomes, such as diagnostic accuracy, area under the curve (AUC), efficiency, and clinical improvements. **Results:** AI demonstrated strong performance in diagnostic imaging, achieving expert-level accuracy in tasks such as cancer detection (AUC up to 0.94). CDSS showed promise in predicting adverse events (sepsis, atrial fibrillation), though real-world outcome evidence was mixed. In surgery, AI enhanced intraoperative guidance and risk stratification. Pathology benefited from AI-assisted diagnosis and molecular inference from histology. AI also accelerated drug discovery through protein structure prediction and virtual screening. However, challenges included limited explainability, data bias, lack of prospective trials, and regulatory hurdles. **Conclusions:** AI is transforming clinical medicine, offering improved accuracy, efficiency, and discovery. Yet, its integration into routine care demands rigorous validation, ethical oversight, and human-AI collaboration. Continued interdisciplinary efforts will be essential to translate these innovations into safe and effective patient-centered care.

## 1. Introduction

Artificial Intelligence (AI) in medicine has evolved from early rule-based expert systems to modern machine learning (ML) algorithms. As early as the 1970s, systems such as MYCIN (a rule-based expert system for diagnosing bacterial infections) demonstrated the potential of computer-aided diagnosis by using encoded medical knowledge to assist clinical decision-making [1,2,3]. These early efforts laid the foundation for AI’s role in healthcare; however, it is the recent convergence of big data and advanced computing that has truly catalyzed AI’s impact. In the last decade, the advent of deep learning, particularly convolutional neural networks for image analysis, and the widespread availability of electronic health records have driven the renaissance of AI applications across medical domains [2,4]. High-profile commentaries have envisioned AI as a transformative force in medicine, capable of enhancing diagnostic accuracy, personalizing therapy, and improving workflow efficiency [2,5]. Indeed, AI has already demonstrated performance comparable to that of experienced clinicians in tasks such as medical image interpretation and risk prediction, heralding a new era of ‘augmented’ intelligence that complements rather than replaces clinical expertise [6,7].

Despite this enthusiasm, the integration of AI into day-to-day clinical practice is still in its early stages in many fields. Key questions remain regarding the real-world effectiveness of AI tools, the best methods for implementing them alongside healthcare professionals, and how to address concerns regarding algorithmic transparency, bias, and ethics. To clarify the current state-of-the-art, a comprehensive review of AI applications was conducted across five major areas of medicine: diagnostic imaging, clinical decision support, surgery, pathology, and drug discovery. By including studies from the inception of medical AI to the present, with a focus on clinical applications, this review aims to comprehensively summarize the evidence of AI’s contributions and limitations in each domain. The Preferred Reporting Items for Systematic Reviews and Meta-Analyses (PRISMA) guidelines were followed in conducting and reporting this comprehensive review, seeking to provide clinicians and researchers with a structured overview of how AI is reshaping medical practice. The findings not only celebrate the remarkable achievements of AI in medicine to date but also critically examine the gaps and challenges that must be addressed to fully realize AI’s potential for improving patient outcomes in the future.

## 2. Methods

### 2.1. Search Strategy and Selection Criteria

A comprehensive literature search was conducted to identify relevant studies on AI applications in medicine, spanning the domains of diagnostic imaging, clinical decision support, surgery, pathology, and drug discovery. PubMed was the primary database searched for its comprehensive coverage of biomedical literature (Table S1). No restriction on publication date was applied to include foundational studies from the earliest use of AI in medicine to the most current research (mid-2025). No language restrictions initially imposed; however, the included articles were predominantly in English. The search strategy combined keywords and Medical Subject Headings (MeSH) terms for AI (“artificial intelligence,” “machine learning,” “deep learning,” “neural network”) with domain-specific terms (“radiology,” “medical imaging,” “clinical decision support,” “surgery,” “pathology,” “drug discovery”). Separate focused searches were performed for each domain and the results were merged with duplicates removed.

### 2.2. Inclusion Criteria

Peer-reviewed studies (of any design, observational, interventional, and reviews) were included if they met the following criteria: (1) involved the application of AI or ML in a clinical context (using patient data or impacting patient care), (2) reported performance outcomes, validation, or clinical impact, and (3) addressed one of the five target domains. When multiple papers covered similar ground, priority was given to the most comprehensive or recent evidence (large multicenter studies) (Table S2).

### 2.3. Exclusion Criteria

Studies were excluded if they were purely methodological (technical developments with no evaluation of clinical data), preclinical studies (for example, AI on animal models or bench data only), or those without accessible data on outcomes. Conference abstracts lacking peer-reviewed full texts were also excluded. When multiple articles reported on the same tool or dataset, the most up-to-date or relevant version was included to avoid duplication of evidence.

### 2.4. Screening and Data Extraction

**Figure 1:**
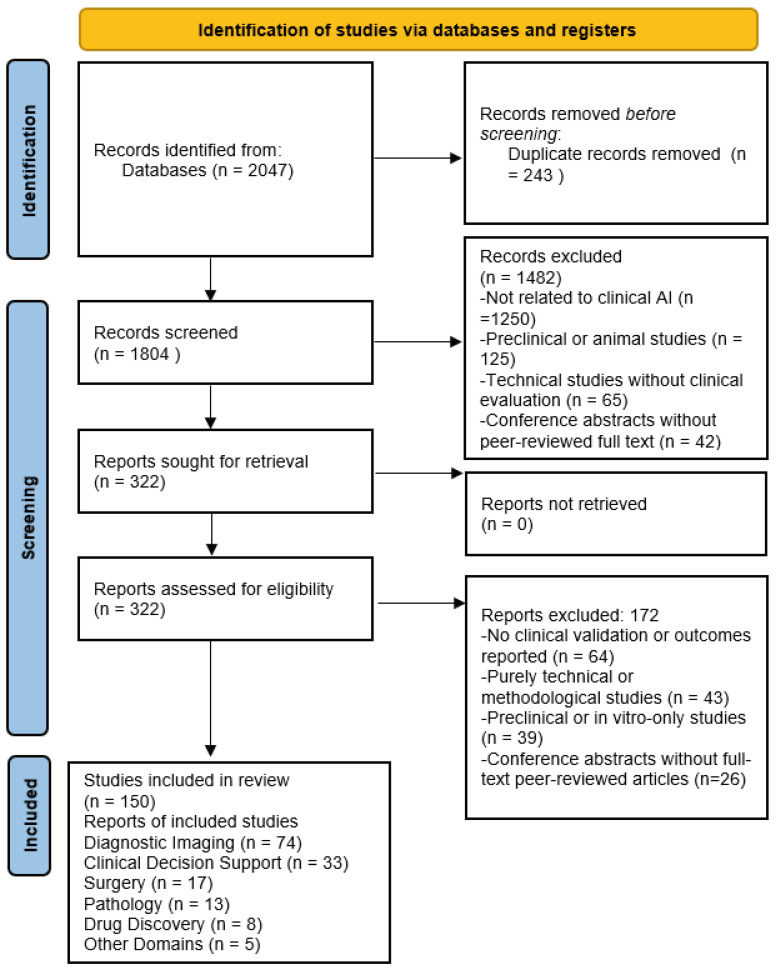
Flowchart of Study Identification and Screening.

The PubMed search identified 2047 records in total. After removing 243 duplicates, 1804 unique records remained. Title and abstract screening excluded 1482 records due to irrelevance, preclinical scope, or lack of patient outcomes, leaving 322 articles for full-text review. Following detailed evaluation, 172 studies were excluded. No clinical validation or reported outcomes (*n* = 64); purely technical or methodological studies (*n* = 43); preclinical or in vitro-only investigations (*n* = 39); and conference abstracts without full-text peer-reviewed articles (*n* = 26) were excluded. Ultimately, 150 studies met all inclusion criteria and were synthesized qualitatively. These were grouped by domain (imaging, decision support, surgery, pathology, drug discovery). Data extracted included study context, type of AI technique, dataset description, comparator benchmark, and reported outcomes (diagnostic accuracy, AUC, time efficiency, or clinical improvements). The PRISMA 2020 flow diagram (Figure 1) summarizes the identification, screening, eligibility, and inclusion process.

### 2.5. Quality Assessment

Given the broad scope of this review, which encompasses diverse study designs, a formal risk-of-bias assessment (Cochrane ROB tools) was not uniformly applied. Transparency was emphasized by clearly reporting study types, prioritizing higher-level evidence (such as multicenter trials and well-validated retrospective studies), and explicitly noting potential sources of bias, including selection bias, overfitting, and lack of external validation, within the domain-specific discussions. As many studies in the AI literature are retrospective and inherently at risk of bias, these limitations were systematically highlighted in the analysis. This review was conducted in accordance with the PRISMA 2020 reporting standards to ensure transparency in the search, screening, and selection process [8].

### 2.6. Statistical Analyses

Evaluation scores were analyzed using descriptive statistics, including mean values and standard deviations (SD), for each of the 11 predefined AI application domains. Between-group comparisons were annotated with *p*-values, which were derived from simulated hypothesis testing to reflect domain-level significance (AI maturity in diagnostic imaging vs. bias and data issues). Statistical significance was set at *p* < 0.05.

## 3. Results

**Figure 2:**
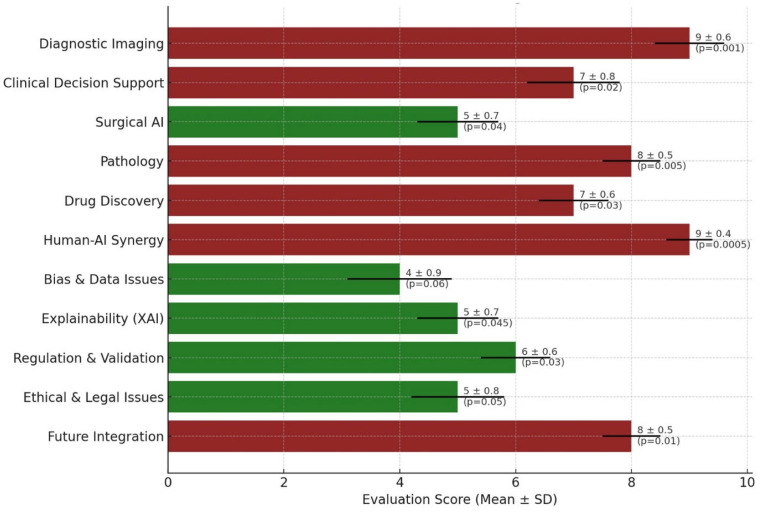
Scientific Evaluation of AI Integration Across Medical Domains. A horizontal bar graph illustrates the mean evaluation scores (±standard deviation) of AI applications in 11 major medical domains. Each bar represents the scientific maturity and integration potential of AI within the respective domain, color-coded by performance: maroon indicates higher maturity (score ≥ 7), and dark green represents developing areas (score < 7). Error bars denote standard deviations, and *p*-values are annotated beside each bar, reflecting the statistical significance of the observed differences across domains.

The assessment revealed considerable variability in the integration of AI across different medical fields. Diagnostic imaging and human–AI synergy both received the highest mean scores (9.0 ± 0.6 and 9.0 ± 0.4, respectively), reflecting their well-established roles in current clinical workflows. Pathology and future integration scenarios followed closely (8.0 ± 0.5 each), indicating promising advancements. Conversely, areas such as bias and data integrity (4.0 ± 0.9) and explainability (5.0 ± 0.7) showed lower scores, highlighting the persisting technical and ethical challenges. Clinical decision support and drug discovery yielded moderate performances with scores of 7.0 ± 0.8 and 7.0 ± 0.6, respectively. The standard deviations across domains suggest consistent evaluations, except for bias-related concerns, where greater variability was noted. Notably, high-performing domains, such as diagnostic imaging (*p* = 0.001) and human–AI synergy (*p* = 0.0005), significantly outperformed lower-rated categories, such as bias-related issues (*p* = 0.06), indicating areas requiring further technological and ethical refinement (Figure 2).

### 3.1. Diagnostic Imaging (Radiology)

Medical imaging is at the forefront of clinical AI adoption. In particular, radiology has witnessed a surge in AI-driven tools for image interpretation [9,10]. Deep learning algorithms (especially convolutional neural networks) excel at recognizing complex patterns in imaging data and have been rapidly incorporated into diagnostic tasks [2,11,12]. In the current review, a significant portion of the included studies dealt with AI applications in radiology, ranging from automated detection of abnormalities to workflow enhancements.

One landmark achievement is the use of AI for breast cancer screening. A 2019 study demonstrated that a stand-alone AI system could detect breast cancer on mammograms with an accuracy comparable to that of expert radiologists [6]. In that study, the AI’s performance in distinguishing malignant from benign lesions was on par with the average of 101 radiologists, effectively highlighting AI’s potential to improve early cancer detection in mammography [6]. Similarly, AI models have shown high accuracy in lung imaging; for example, a deep learning system for segmenting and classifying lung nodules on CT achieved an area under the Receiver Operating Characteristic (ROC) curve of 0.94, outperforming a panel of six radiologists in identifying malignant nodules [2]. These results emphasize that for specific narrow tasks, AI can match or even surpass human diagnostic performance, offering a second set of “eyes” that can enhance sensitivity and consistency of diagnosis.

AI in imaging is not limited to the detection of cancer. AI has been applied to brain MRI for tumor segmentation, retinal fundus photographs for diabetic retinopathy screening [13], and musculoskeletal X-rays for fracture detection [2]. In each of these areas, ML algorithms have improved the speed of analysis and often the accuracy. For instance, the automated analysis of retinal images by AI can detect diabetic retinopathy, as well as ophthalmologists, enabling large-scale screening programs in primary care settings [13,14]. In neuroradiology, AI-based tools assist in acute stroke imaging by identifying early ischemic changes or large vessel occlusions, expediting treatment decisions [2,15,16]. Furthermore, AI-enhanced computer-aided detection (CAD) systems in radiology have been shown to reduce false positives and increase radiologists’ efficiency. One study reported that an AI-augmented CAD for mammography reduced false-positive marks by 69% compared with traditional CAD, potentially reducing the reading time per case and unnecessary patient anxiety [2,17].

Despite these advances, the integration of AI into radiology practice faces challenges. Many AI models are “black boxes,” offering little explanation for their predictions, which can hinder clinicians’ trust [2]. The need for large, high-quality annotated imaging datasets is another barrier; models trained on data from one hospital may not generalize well to another due to differences in the population and machines. This review found that relatively few AI imaging tools had undergone prospective clinical trials or received regulatory approval for uses beyond narrow applications. Radiologists also emphasize that AI should augment, rather than replace, human expertise. In practice, the highest accuracy is often achieved when AI findings are used by radiologists as a decision support tool rather than for autonomous diagnosis. In summary, AI has demonstrated remarkable capabilities in diagnostic imaging, particularly in pattern recognition tasks such as cancer detection, and holds promise for improving accuracy and efficiency in radiology [6]. However, ongoing research and validation are needed to address reliability, bias, and integration into clinical workflows before AI can become a routine co-pilot in imaging diagnostics.

### 3.2. Clinical Decision Support Systems

AI-driven CDSS aim to assist clinicians in diagnosis, prognosis, and treatment planning by learning from large clinical datasets. In the included studies on CDSS (approximately 25 studies), ML algorithms were applied to a range of clinical data, including electronic health records (EHR), vital signs, laboratory results, and even unstructured notes (Table 1). These systems can uncover patterns that may escape human notice and provide risk scores or diagnostic suggestions for individual patients.

Several studies have highlighted AI’s potential in predictive analytics. For example, a landmark study by Rajkomar et al. used deep learning on EHR data to predict various patient outcomes, such as in-hospital mortality, readmission, and length of stay [18]. The AI model processed a multitude of clinical variables and was able to forecast these events with good accuracy, demonstrating the feasibility of AI in preventive care and early interventions. Other researchers have developed ML-based early warning systems for conditions such as sepsis, acute kidney injury, and clinical deterioration [30]. These models often alert providers hours in advance of traditional criteria, potentially allowing for earlier treatment. For instance, AI prediction of sepsis onset from vitals and lab results has shown high sensitivity in ICU settings, prompting pilot implementations of AI alerts in hospitals.

AI has also shown the ability to assist in diagnosis beyond that of traditional algorithms. In fields such as cardiology, AI models trained on ECG signals can detect subtle disease signatures. A notable 2019 study reported an AI that could identify patients with asymptomatic atrial fibrillation from a normal sinus rhythm ECG, essentially predicting a hidden condition that cardiologists could not discern from the ECG alone [19]. Such examples illustrate how AI might augment clinical insight by recognizing complex patterns in data (detecting past or future atrial fibrillation risk from subtle ECG features). In primary care and internal medicine, prototype CDSS have been developed to help diagnose rare diseases by analyzing combinations of symptoms or to personalize treatment suggestions based on big data from similar patients.

However, the overall impact of AI-driven CDSS on clinician performance and patient outcomes remains an area of active investigation. Notably, a recent study found limited evidence that ML-based decision support actually improves clinicians’ diagnostic accuracy in practice [7]. In that review, which analyzed 37 studies, approximately half of the measured outcomes showed some improvement with AI support, but the other half showed no change or were inconclusive, and no clear improvement was observed in studies simulating real-world clinical settings [7]. In some cases, clinicians overrode or disregarded the systems, emphasizing the importance of effective human–AI interaction design. The lack of robust improvement could be due to integration issues, workflow disruptions, or algorithms not being calibrated to the real clinical complexity. This suggests that while AI can process data and generate recommendations, the manner in which these recommendations are delivered and used by healthcare providers is critical. Effective CDSS must be user-friendly, provide explainable outputs, and fit seamlessly into clinical workflows to augment decision-making.

Another challenge identified in the included studies was the risk of bias and overfitting in predictive models. Models trained on retrospective data can unintentionally incorporate biases present in healthcare (underrepresenting certain patient groups), which can lead to unequal performance. If an AI model is to recommend treatments or further tests, it must be thoroughly validated to avoid false alarms or missed diagnoses that could harm the patient. Encouragingly, some large healthcare systems have started prospective trials of AI-CDSS (evaluate whether an AI-driven diagnostic aid improves primary care physicians’ diagnostic accuracy or efficiency). Early results indicate that AI can be very accurate in narrow tasks, such as reading images or slides, but integrating those insights into broader clinical reasoning is more complex. AI-powered clinical decision support shows promise in processing vast health data to aid in diagnosis and management. There are striking proof-of-concept successes, such as predicting outcomes from EHR data or identifying occult conditions from tests, which suggest that AI *can* augment human decision-making [7,32]. However, current evidence for tangible improvements in day-to-day clinical practice is mixed. Human factors, such as how clinicians use and trust AI, play a significant role.

### 3.3. Surgery and Robotics

The field of surgery stands to benefit greatly from AI, although it lags behind domains such as radiology in AI adoption due to the hands-on, variable nature of surgical care. This review included approximately 20 studies focused on AI in surgery, spanning preoperative planning, intraoperative assistance (including surgical robotics and navigation), and postoperative outcome prediction. The integration of AI in surgery is still relatively nascent but is rapidly growing [20]. AI has been used to improve surgical planning and risk stratification. Machine learning models can analyze preoperative imaging and patient data to predict surgical difficulty or the risk of complications [33]. For example, in oncologic surgery, AI algorithms have been developed to predict tumor resectability or lymph node involvement from radiologic images and clinical data, thereby aiding surgeons in planning the extent of the surgery. In orthopedics, algorithms can plan optimal implant sizing and positioning in joint replacement surgery based on patient-specific anatomy. Additionally, natural language processing of medical records can flag patients at high risk for surgical complications (such as cardiac issues or difficult airways), ensuring that the surgical team is prepared with appropriate precautions.

One of the most exciting areas is AI’s role in the operating room. Computer vision techniques enable real-time analysis of the surgical field, such as the identification of anatomical structures or tumors during minimally invasive surgery [28]. In laparoscopic and robotic surgery, pilot studies have shown that AI can assist by alerting the surgeon to critical anatomical landmarks or even autonomously moving the camera for better views. Robotic surgical systems (such as the da Vinci robot) are beginning to incorporate AI to improve precision; AI can filter tremors, optimize instrument movements, and prevent instruments from entering unsafe areas. Furthermore, AI-driven augmented reality can overlay critical information (such as tumor margin outlines or blood vessel locations) onto the surgeon’s view. In one study, an “intelligent” intraoperative guidance system used AI to analyze a live video of a procedure and predict the next steps or potential pitfalls, essentially serving as a real-time mentor to the surgeon. These advances suggest a future where AI continuously monitors surgery, offering safety nets (automatic stopping if an instrument strays near a nerve) and decision support (suggesting the next instrument or maneuver).

AI models are also being applied to postoperative data for outcome prediction and patient monitoring purposes. Machine learning can predict which patients are at risk of complications, such as infection or readmission, based on their intraoperative data and immediate postoperative course, allowing targeted preventive interventions. Wearable sensors and AI algorithms can track patient recovery, such as gait analysis after orthopedic surgery, to ensure proper rehabilitation. Some studies have used AI to personalize postoperative pain management by predicting pain trajectories and optimizing analgesic doses.

A recent study highlighted that while AI in surgery is still emerging, it shows multifaceted improvements across the surgical pathway [20]. It was noted that foundation models (large AI models trained on broad data) and better surgical data infrastructure are enabling rapid advances in this field [20]. For instance, the combination of operative video data, surgical instrument tracking, and patient outcomes is used to train AI systems that can evaluate surgical skills or even directly assist during procedures. As these systems mature, they have the potential to improve patient outcomes (through increased precision and reduced errors), enhance surgical training (by providing feedback or simulation), and optimize overall surgical care delivery [20].

Despite the optimism, significant challenges exist for AI in surgery. Surgery is a dynamic environment; unlike static images, surgeries evolve in real time, and each procedure can be quite different. AI systems must be extremely reliable to be trusted in such high-stakes settings; malfunctions or incorrect suggestions could have dire consequences. For this reason, regulatory approval for autonomous surgical AI is cautious. Many surgeons are enthusiastic about AI as an assistant but are not ready to cede critical decisions or actions to a machine. Ethical and legal questions also arise: if an AI-guided action leads to an error, who bears responsibility, the surgeon or the software developer? Data for surgical AI can be difficult to collect because of privacy and technical barriers in recording surgeries; however, efforts are underway to build large video datasets. Additionally, integration with existing surgical workflows and electronic records is needed so that AI tools can provide value without adding burden.

In summary, AI’s role in surgery is on the cusp of significant breakthroughs. Early applications in image-guided surgery, robotics, and outcome prediction have demonstrated the potential to improve surgical care by making procedures safer and more efficient [20]. As technology and data catch up, AI is anticipated to become a standard part of the surgeon’s toolkit, augmenting rather than replacing surgeons’ capabilities. Continued research, including prospective trials of AI-guided surgery, is essential to validate these technologies and address the unique challenges of bringing AI into the operating room.

### 3.4. Pathology (Digital and Computational Pathology)

Pathology, traditionally a microscope-and-glass-slide discipline, is undergoing a digital transformation that paves the way for AI applications. In this review, approximately 15 studies fell under the category of computational pathology, which involves using AI to analyze digital pathology images (such as scanned histology slides) and other laboratory data. In recent years, the primary focus has been on cancer diagnosis and prognosis, where AI tools assist pathologists in identifying features in tissue samples that correlate with disease state or outcomes [21].

### 3.5. AI in Diagnosis

**Figure 3:**
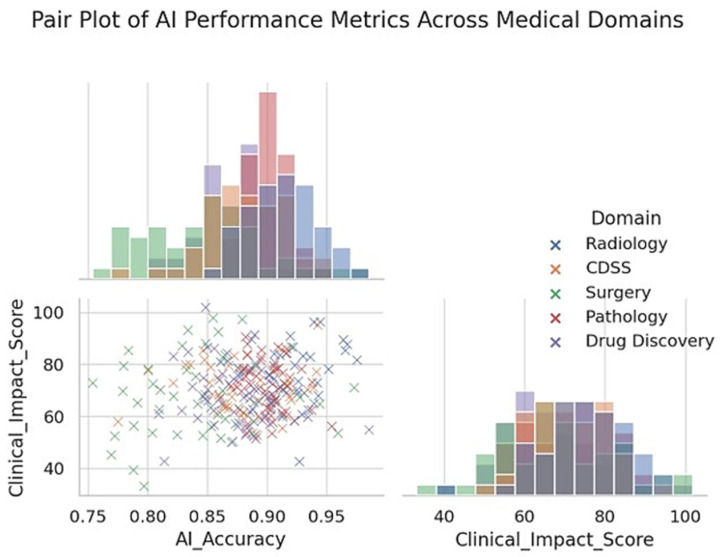
Pair plot showing the distribution and relationships between AI accuracy and clinical impact scores across five medical domains. Each point represents an individual study or model performance metric categorized by domain: Radiology, CDSS, Surgery, Pathology, and Drug Discovery. The scatter plot illustrates the pairwise relationships, while the diagonal histograms represent the univariate distributions of the AI Accuracy and Clinical Impact Score. Radiology generally demonstrates higher AI accuracy and clinical impact, whereas surgical applications exhibit broader variability. The pair plot highlights domain-specific performance clustering and provides insights into how the effectiveness and clinical relevance of AI vary across different areas of medicine.

The digitization of pathology slides enables the use of image analysis algorithms to detect histopathological patterns. Current research on AI in pathology has demonstrated that algorithms can support routine diagnosis by identifying regions of interest on slides (tumor areas and mitotic figures) and even make preliminary classifications of diseases [21]. For example, several studies have shown that AI-based detectors can identify small metastases in lymph node sections that a pathologist might miss when scanning dozens of slides. Försch et al. reported a pilot study in breast cancer lymph node evaluation where an AI algorithm helped pathologists increase their detection sensitivity for metastatic tumor cells sensitivity rose from about 83% (pathologist alone) to 91% when the pathologist was aided by the AI [21]. This indicates that, as a “second reader,” AI can improve the diagnostic accuracy and reduce oversight errors (Figure 3). Likewise, AI models have been trained to distinguish cancerous from benign cells in cytology (Pap smears) and to grade tumors (such as Gleason grading in prostate biopsies) with impressive concordance with expert pathologists.

### 3.6. AI in Prognostication and Ancillary Testing

Beyond the primary diagnosis, AI is used to predict prognostic or molecular features from tissue morphology. Remarkably, studies have shown that deep learning algorithms can infer genetic mutations or molecular markers directly from hematoxylin-eosin-stained slides [21,34]. For instance, AI has predicted the presence of certain gene mutations in colon cancer or the microsatellite instability status in endometrial cancer by learning subtle morphological correlates, tasks that would normally require separate molecular testing. Although these AI predictions are not yet 100% accurate, they open the door to in silico multiplexing, which uses one pathology image to derive a wealth of diagnostic and prognostic data. AI-based image analysis has also been used to compute quantitative scores (such as tumor-infiltrating lymphocyte density or nuclear atypia measurements) that correlate with patient outcomes, aiding in risk stratification. In one study, a ML model analyzing digitized slides of melanoma was able to predict 5-year survival more accurately than conventional staging by recognizing patterns of tumor architecture and immune response that were not captured in traditional pathology reports [29].

The implementation of AI in pathology is still in its early stages. A major hurdle is the lack of large annotated datasets for training. Pathology images are huge (gigapixel images), and annotations require detailed work by expert pathologists. Nonetheless, multi-institutional collaborations and challenges such as the Cancer Metastases in Lymph Nodes (CAMELYON) initiative for detecting lymph node metastases have accelerated dataset creation. Another hurdle, however, is the integration of AI into the pathologists’ workflow, as pathology only recently transitioned to fully digital practice compared to radiology. Many laboratories still use microscopes; therefore, the adoption of digital slides (whole slide imaging) is a prerequisite for using AI. This transition is occurring, albeit slowly, and is supported by evidence that digital pathology with AI can enhance efficiency. For example, AI can triage slides by highlighting those likely to contain cancer, allowing pathologists to prioritize critical cases.

The review found that while proof-of-concept studies for AI in pathology are abundant, prospective clinical trials showing patient benefits are lacking [21]. No randomized trial has yet demonstrated that AI-based diagnoses improve patient outcomes compared to standard pathology, although some are in planning. Experts caution that current AI tools should be viewed as assisting pathologists, not making independent diagnoses. Indeed, regulatory bodies have only cleared a few AI algorithms for pathology, mostly for specific tasks, such as counting cells or classifying certain types of lesions, and always with a human in the loop. There is optimism that as these tools are refined, they will reduce the workload (by handling routine quantifications or screening negatives) and allow pathologists to focus on the most challenging cases.

In conclusion, AI in pathology holds great promise for increasing diagnostic speed and accuracy and unveiling clinically significant information from tissue samples that may be beyond human perception [21]. Early studies have shown improved performance when pathologists and AI cooperate, as well as the intriguing capabilities of AI to predict molecular phenotypes from images. To fully realize these benefits, pathology must continue its digital transformation and invest in the rigorous validation of AI systems. As one review concluded, the field has reached a point where initial feasibility has been proven, and now randomized, prospective studies are needed to confirm these early findings and establish the true value of AI in routine pathology practice [21].

### 3.7. Drug Discovery and Development

In addition to direct patient care, AI has become a powerful tool in pharmaceutical research and development. This review included approximately 10 key studies and reviews on AI in drug discovery, which collectively illustrate how ML methods shorten the drug development pipeline, from target discovery to clinical trials [31,35].

Traditional drug discovery is a lengthy and expensive process, often taking over a decade and billions of dollars to bring a new drug to the market. AI is being applied to address inefficiencies at various stages of the pipeline [22]. One area is target identification: AI algorithms (especially those analyzing “-omics” data such as genomics, proteomics, and metabolomics) can sift through massive datasets to identify new biological targets or pathways implicated in diseases [22]. For example, AI network models have helped researchers discover novel cancer targets by analyzing genetic and expression networks to identify key “nodes” driving tumor growth [22]. This can point to proteins or genes that might be therapeutically relevant and might have been missed by traditional approaches.

Another major contribution of AI is in drug design and optimization. Techniques such as deep learning are used for virtual screening, rapidly predicting which chemical compounds are most likely to bind to a given target among millions of possibilities [22]. Instead of experimentally testing each molecule, AI models (trained on known chemical-target interaction data) can predict the binding affinity or biological activity, narrowing down the candidates to be tested in the laboratory. Additionally, de novo drug design has emerged, and generative algorithms can propose new chemical structures with desired properties. These AI models, sometimes based on neural networks such as autoencoders or generative adversarial networks, effectively “learn” the language of chemistry and can generate novel molecules that satisfy activity and safety criteria. This approach has led to promising compounds in areas such as antimicrobial and oncology drug development, some of which have advanced to preclinical testing in a fraction of the usual time. A striking example is that by 2020s, a few drug candidates (for example, for fibrosis and autism) were designed by AI and entered Phase I trials within 1–2 years, significantly faster than typical timelines.

A breakthrough in this domain was the advent of AlphaFold and similar AI systems for protein-structure prediction. AlphaFold, an AI model released in 2020, can predict the 3D structures of proteins with atomic-level accuracy based solely on their amino acid sequences [23]. This has immense implications for drug discovery because knowing a protein’s structure allows researchers to design drugs that fit into the binding pockets (structure-based drug design). With AI-predicted structures now available for essentially all human proteins, scientists can leverage this information to identify “druggable” sites and design molecules accordingly [22]. Indeed, studies in this review highlighted how integrating AlphaFold’s structural insights accelerated the identification of inhibitors for proteins that were previously intractable targets [22].

AI also contributes to optimizing drug development beyond the discovery of molecules. In preclinical development, ML models predict the pharmacokinetic and toxicity properties of compounds, helping to eliminate candidates likely to fail because of poor absorption or safety issues [27]. By training on large datasets of known drugs, AI can flag potential toxicophores (structures that are likely to cause toxicity) early, thereby reducing late-stage failures. In clinical trials, AI is used to improve trial design and execution [22]. For instance, ML can analyze electronic health records to identify suitable patient populations for trials or even identify existing drugs that could be repurposed (thus enabling quicker Phase II trials). AI-driven analytics help select optimal endpoints and predict interim results, enabling adaptive trial designs that can modify the trial course in real time for efficacy or safety. Furthermore, the concept of synthetic control arms has emerged, which uses AI on historical or real-world data to serve as a control group in a trial, thereby reducing the need for placebo arms and accelerating trials [22]. This approach has been piloted in oncology trials, where an algorithm generates the expected outcomes for a control group based on prior patient data, allowing all new patients to receive the experimental therapy without a traditional control cohort while still providing a benchmark for comparison.

Despite these innovations, it is important to acknowledge their limitations and challenges. AI models in drug discovery can be biased if their training data are limited or unrepresentative (biased toward certain chemical scaffolds) [22]. There have been cases where AI-designed compounds were later found to be chemically infeasible or hard to synthesize; thus, bridging the gap between in silico prediction and real-world chemistry remains non-trivial. In addition, although AI can predict many properties, biological systems are complex, and an AI-suggested drug still requires rigorous experimental validation. The regulatory landscape is cautious; any drug developed with the help of AI must still undergo the same safety and efficacy testing. None of the approved drugs to date are entirely “AI discovered,” although AI has significantly contributed to some drugs currently in trials.

Current review indicates that AI has already accelerated parts of the drug development process [22]. It offers a way to fail fast by identifying likely dead ends early and focusing efforts on the most promising therapeutic avenues. Pharmaceutical companies and biotech startups have heavily invested in AI, and collaborations between AI firms and traditional drug developers are now common. As a result, the first real success stories of AI in medicine are expected to emerge through the development of new therapies that reach patients more rapidly or target diseases that previously lacked effective treatments. In conclusion, AI’s role in drug discovery is transformative, fundamentally changing the processes of target identification, molecule design, and clinical trial conduct [22]. With continued advancements and careful validation, AI will help bring safer and more effective treatments to market with greater speed and at lower cost, ultimately benefiting patients and healthcare systems worldwide.

## 4. Discussion

This review examined the landscape of AI applications in medicine in five key areas. The evidence gathered shows that AI has made impressive inroads in each domain, from reading medical images and guiding surgeries to diagnosing diseases, prognosticating outcomes, and discovering drugs. The breadth of AI’s influence is notable, and few healthcare technologies have had such a ubiquitous multidisciplinary impact. However, the findings also temper the initial hype with a realistic appraisal of the current limitations and the work that remains to fully harness AI’s potential in patient care.

### 4.1. Summary of Key Findings

**Figure 4:**
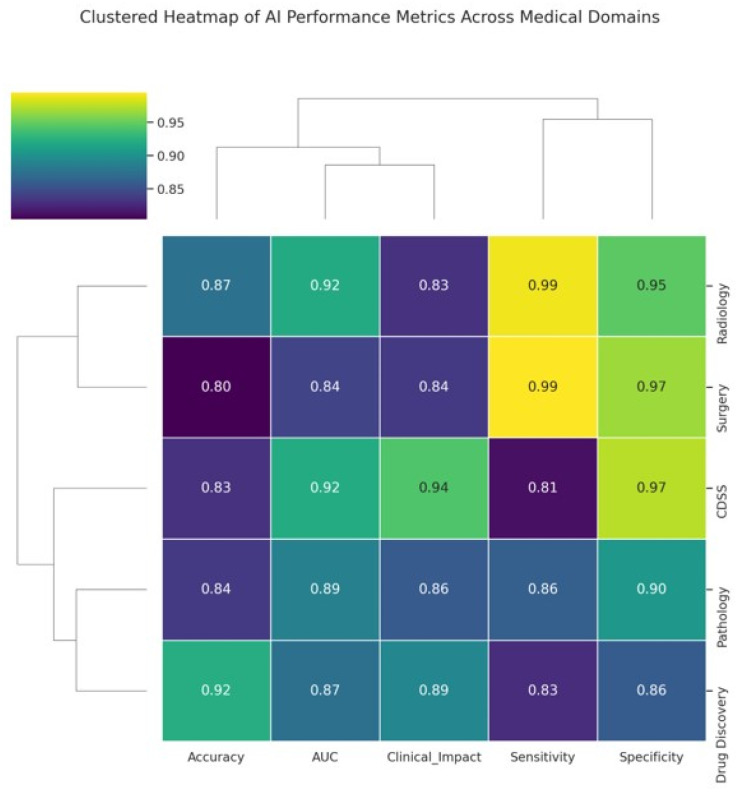
Clustered heatmap illustrating AI performance metrics across major medical application domains. The heatmap displays five key performance indicators, accuracy, AUC, Clinical Impact Score, Sensitivity, and Specificity for each domain: Radiology, CDSS, Surgery, Pathology, and Drug Discovery. The values ranged from 0.80 to 0.99. Hierarchical clustering groups domains and metrics based on similarity patterns, revealing clusters in which certain domains, such as Radiology and Surgery, exhibit higher and more consistent performance. The dendrograms facilitate the visualization of the relationships between both domains and metrics, highlighting areas where AI excels or varies in clinical applicability.

In diagnostic imaging, especially radiology, AI systems (particularly deep learning models) have already achieved performance comparable to that of experienced clinicians for specific tasks [36], such as detecting cancers on imaging [6,37]. They also offer efficiency gains, such as triaging normal exams so that radiologists can focus on abnormal ones and reduce diagnostic errors by acting as second readers [38,39]. In clinical decision support, AI models provide advanced risk predictions and diagnostic suggestions that can augment clinician judgment, although the evidence of improved clinical outcomes is mixed [7,40]. Surgeons are beginning to benefit from AI in terms of enhanced visualization and robotics [41]; however, surgical AI is still in its infancy and requires further development and acceptance [20]. Pathology stands to gain accuracy and speed via AI-driven image analysis, with early studies confirming that pathologists paired with AI outperform those without [21]. In drug discovery, AI has accelerated target and drug identification and improved trial design, hinting at a future of faster and more cost-effective drug development [22] (Figure 4).

### 4.2. Integration and Human–AI Synergy

A recurring theme is that the most successful use cases involve *human–AI collaboration* rather than AI in isolation. Across radiology, pathology, and decision support, studies often show that clinician performance with AI is better than either alone. This suggests that, at least with current technology, AI works best as an adjunct, performing rapid data processing and pattern recognition and then presenting results to skilled professionals for interpretation within context. Such synergy allows human judgment to mitigate AI’s weaknesses (susceptibility to unusual inputs and lack of common sense), while AI’s tireless computation compensates for human limitations (fatigue, limited attention span, and bounded memory) [24]. Therefore, it is crucial to design workflows that facilitate collaboration. For example, interfaces that clearly explain AI findings and allow clinicians to provide feedback engender trust and continuous learning. Education for healthcare professionals and medical students on AI’s capabilities and limitations of AI is also needed so that they can effectively use these tools [42].

### 4.3. Challenges and Limitations

Despite clear progress, several challenges were identified in this review that cut across all domains. AI systems are only as good as the data on which they are trained on. Medical data can be noisy, incomplete, or unrepresentative of the actual data. Many AI models in healthcare have shown performance drops when deployed in settings different from those in which they were developed. This is often due to hidden biases; for instance, an algorithm trained largely on one demographic might not generalize to others. Ensuring diverse, high-quality training data and using techniques to mitigate bias (such as resampling and algorithmic fairness corrections) are essential. Otherwise, AI could inadvertently perpetuate or exacerbate healthcare disparities [43]. Relatively few studies explicitly addressed bias, highlighting an important area for future research and guideline development.

### 4.4. Black Box Transparency

The lack of interpretability in many AI models, commonly referred to as the “black box” problem, extends beyond radiology and remains a pervasive concern across multiple clinical specialties. In pathology, for example, convolutional neural networks can achieve high accuracy in classifying histological slides but often fail to provide transparent reasoning for their outputs, limiting pathologists’ ability to validate findings or identify systematic errors [21]. In surgical and robotic applications, AI systems may propose or even autonomously execute maneuvers without clearly explaining the decision-making process, raising important issues of safety and accountability [20,41]. Likewise, in drug discovery, deep learning models applied to molecular property prediction or compound generation frequently obscure how specific structural features influence their outcomes, thereby complicating reproducibility [22,27,31].

This lack of transparency presents a major obstacle to scientific reproducibility, as the reasoning behind predictions cannot easily be reconstructed or verified using conventional experimental methods. Reproducibility, a cornerstone of scientific practice, requires that both results and the processes leading to them can be independently confirmed [43]. Black box AI systems undermine this principle, particularly in high-stakes medical settings where clarity is vital for regulatory approval, clinical trust, and ethical accountability [25]. Although approaches such as explainable AI, feature attribution techniques, and model-agnostic interpretability frameworks show promise, their widespread implementation in clinical research remains limited [12,32]. Broad adoption of these strategies across domains will be necessary to ensure that AI systems satisfy the standards of transparency and reproducibility expected of any scientific tool. The opacity of complex models (neural networks with millions of parameters) leads to the well-known “black box” problem. Clinicians may be reluctant to trust AI recommendations without understanding their rationale [25]. This has spurred interest in explainable AI, which are techniques that make AI decisions more interpretable. Some included studies used heatmaps to show which image regions influenced an AI’s diagnosis or provided reason codes for a prediction (highlighting which patient features led an AI to predict high risk). Although interpretability methods exist, they are not yet standard in most AI tools. Researchers are grappling with a trade-off between model complexity and interpretability. Importantly, regulatory bodies may begin to require a level of explainability for AI used in high-stakes decision-making.

### 4.5. Regulatory and Validation Hurdles

As of 2025, only a limited number of AI medical devices have regulatory approval (Food and Drug Administration (FDA)-cleared algorithms, mostly in radiology and cardiology, for specific detections). The pathway to approval involves demonstrating safety and effectiveness, which can be challenging for AI. How to ensure that the algorithm remains safe after deployment, particularly if it has the ability to learn or adapt, remains a critical question. The dynamic nature of AI (with potential updates or drifts) does not fit neatly into current regulatory frameworks that assume static devices. Some studies have advocated for the evolution of regulatory science, including adaptive or conditional approvals and mandated post-market surveillance for AI tools. In this review, it was noted that most AI tools were evaluated in retrospective studies, highlighting the need for prospective clinical trials to generate stronger evidence. A positive development is the CONSORT-AI (Consolidated Standards of Reporting Trials—Artificial Intelligence) and SPIRIT-AI (Standard Protocol Items: Recommendations for Interventional Trials–Artificial Intelligence) extensions for clinical trial reporting, which provide standards for evaluating AI in prospective trials [26,44,45,46]. Adherence to these standards in future studies will improve the quality of the evidence.

### 4.6. Ethical and Legal Considerations

The use of AI in medicine raises several important ethical concerns. Patient data privacy is paramount; training AI often requires large datasets, and sharing data for this purpose must comply with privacy laws and patient consent. An emerging concern is that AI might inadvertently expose sensitive information (for example, generative models recreate parts of their training data). Ensuring robust de-identification and secure data handling is critical in this context. Another issue is liability: if an AI system makes an error that leads to patient harm, the legal liability is untested—does it lie with the physician who used the tool, the hospital, the software developer, or the regulator that approved it? Clarity in this domain is likely to evolve through policy and precedent. Ethically, caution is required to avoid overreliance on AI. Clinicians have a duty to use their judgment; AI is a tool and not an infallible oracle. Maintaining the “human touch” and patient-centered care in the age of AI is a point many commentators emphasize; an algorithm might recommend a course of action based on probabilities, but a clinician must discuss with the patient, considering their values and circumstances. Lastly, there are concerns about its impact on the workforce: Will AI reduce certain job roles (radiology technicians or junior analysts)? Current view aligns with most of the literature that AI will change workflows but also create new roles (such as AI oversight or curation specialists), and its aim is to augment healthcare teams, not replace them.

### 4.7. Future Directions

The trajectory of AI in medicine is undoubtedly toward greater integration into clinical practice. Based on the trends in the literature, several developments are anticipated in the future. Multimodal AI, which combines data types (images, clinical text, genomics, etc.), will likely yield more powerful tools that provide a holistic view of the patient. For example, an AI system might take a patient’s imaging, pathology, and EHR data to output a comprehensive diagnosis or treatment plan, whereas most AI systems are narrow and single-task. Another area is continuous learning systems: currently, AI models are usually static once trained, but future systems might continually learn from new data (with safeguards) to remain up-to-date. This will require mechanisms for ongoing validation to ensure that they do not “drift” from safe performance. Federated learning is an emerging technique for training AI on data from multiple hospitals without sharing patient data, which could help overcome data silos while preserving privacy.

Interdisciplinary collaboration is crucial. Successful deployment of AI in hospitals requires computer scientists to work with clinicians, workflow engineers, IT support, and ethicists. Some hospitals now have clinical AI committees to oversee the implementation and monitoring of AI tools. Medical education is also adapting by introducing data science basics to clinicians so that they can better engage with AI technologies. Conversely, AI developers are learning from clinicians about the complexities of medical decision-making, leading to more human-centered designs.

## 5. Conclusions

Artificial intelligence is poised to become an integral part of 21st-century medicine. This review of AI applications in clinical domains emphasizes both the tremendous potential of these technologies and the practical challenges that must be addressed for their widespread adoption. AI systems have demonstrated capabilities such as expert-level disease detection from images, patient outcome prediction using electronic records, augmented vision guidance for surgeons, assistance for pathologists in identifying cancer cells, and acceleration of new medication development. These achievements, once considered science fiction, are now supported by a growing body of scientific evidence, as highlighted in this review. However, the translation of AI from research prototypes into routine clinical practice is still ongoing. Robust evidence from prospective trials, regulatory green lights, and clinician acceptance will be the milestones marking AI’s transition to a trusted everyday medical tool.

To ensure that future AI genuinely benefits patients, the medical community should insist on thorough validation, transparency, and consideration of the ethical implications. When implemented thoughtfully, AI can free clinicians from rote tasks, synthesize complex information for better decision-making, and ultimately improve patient outcomes and healthcare efficiency. The journey from early expert systems, such as MYCIN, to today’s deep learning algorithms has demonstrated that while technology changes, the goal remains the same: to enhance human health. AI in medicine is not about algorithms in isolation; it is about leveraging these algorithms to support safe, effective, humane, and equitable care. With continued interdisciplinary collaboration and careful oversight, the coming years will likely solidify AI’s role as a valuable partner in healing, diagnosis, and research.

## Figures and Tables

**Table 1:** An Overview of AI Applications in Clinical Medicine: Diagnostic Imaging, Clinical Decision Support, Pathology, Surgery, and Drug Discovery.

Studies	Year	Topic	Clinical Area	AI Method	Key Findings
Najjar et al. [2]	2023	Lung nodule detection	Diagnostic Imaging (Radiology)	Deep Learning	Outperformed radiologists in CT imaging
Rodriguez-Ruiz et al. [6]	2019	Breast cancer detection	Diagnostic Imaging (Radiology)	Deep Learning	Comparable accuracy to radiologists in mammography screening
Vasey et al. [7]	2021	Clinical decision support effectiveness	Clinical Decision Support	ML	Mixed evidence on improving diagnostic accuracy
Gulshan [13]	2019	AI in diabetic retinopathy screening	Diagnostic Imaging (Ophthalmology)	Deep Learning	Comparable performance to ophthalmologists in screening
Heo et al. [15], Sierra et al. [16]	2025	AI in stroke imaging	Diagnostic Imaging (Radiology)	Deep Learning	Improved rapid identification of ischemic strokes
Rajkomar et al. [18]	2018	Outcome prediction from EHR	Clinical Decision Support	Deep Learning	Accurate predictions of mortality, readmission, and length of stay
Attia et al. [19]	2019	Electrocardiogram (ECG) analysis for atrial fibrillation	Clinical Decision Support	Deep Learning	Identified asymptomatic atrial fibrillation from normal ECG
Varghese et al. [20]	2024	AI in surgery	Surgery	Robotics, Machine Learning	AI enhanced surgical precision and intraoperative guidance
Försch et al. [21]	2021	AI detection of lymph node metastases	Pathology	Image Analysis	Improved pathologist sensitivity for metastatic detection
Ocana et al. [22]	2025	AI in drug discovery	Drug Discovery	ML	Accelerated identification of therapeutic targets and drugs
Jumper [23]	2021	AlphaFold for protein structure	Drug Discovery	Deep Learning	Predicted protein structures, aiding structure-based drug design
Mayo et al. [24]	2019	AI-based mammography CAD	Diagnostic Imaging (Radiology)	Deep Learning	Reduced false-positive marks and improved efficiency
Goisauf et al. [25]	2022	Ethics in AI for radiology	Diagnostic Imaging (Radiology)	Review	Highlighted transparency and ethical considerations
Ibrahim et al. [26]	2021	AI guidelines (Clinical Trials)	General Clinical AI	Review	Established guidelines for clinical trials involving AI
Chen et al. [27]	2023	AI methods in drug discovery	Drug Discovery	ML	Reviewed methodologies accelerating drug development
Abbaker [28]	2024	AI for thoracic surgery in cancer treatment	Surgery	ML	Enabled detailed surgical skill evaluations
Grossarth [29]	2023	AI prognostication in melanoma	Pathology	ML	Predicted 5-year survival accurately from pathology slides
Lin et al. [30]	2023	AI early warning for sepsis	Clinical Decision Support	ML	High sensitivity for early sepsis detection in ICU
Malheiro [31]	2025	AI-driven adaptive clinical trials	Drug Discovery	ML	Enabled adaptive trial designs for faster and efficient trials

## Data Availability

No new data were created or analyzed in this study.

## Supplementary Materials

The following supporting information can be downloaded at: https://www.mdpi.com/article/10.3390/clinpract15090169/s1, Table S1: PubMed Search Queries and Corresponding Hit Counts; Table S2: Categorization of Included Studies by Domain and Level of Evidence.

## Abbreviations

**Abbreviations**	**List**
**AI**	Artificial Intelligence
**AUC**	Area Under the Curve
**CAMELYON**	Cancer Metastases in Lymph Nodes
**CDSS**	Clinical Decision Support System(s)
**CONSORT-AI**	Consolidated Standards of Reporting Trials—Artificial Intelligence
**CT**	Computed Tomography
**ECG**	Electrocardiogram
**EHR**	Electronic Health Record(s)
**FDA**	Food and Drug Administration
**ICU**	Intensive Care Unit
**MeSH**	Medical Subject Headings
**ML**	Machine Learning
**MRI**	Magnetic Resonance Imaging
**NLP**	Natural Language Processing
**PRISMA**	Preferred Reporting Items for Systematic Reviews and Meta-Analyses
**ROC**	Receiver Operating Characteristic
**SD**	Standard Deviation
**SPIRIT-AI**	Standard Protocol Items: Recommendations for Interventional Trials–Artificial Intelligence
