# Supplementary material for: Artificial Intelligence in Clinical Medicine: Challenges Across Diagnostic Imaging, Clinical Decision Support, Surgery, Pathology, and Drug Discovery
Source: Clin Pract. 2025 Sep 16;15(9):169. doi: 10.3390/clinpract15090169 (PMC12468291; doi:10.3390/clinpract15090169)
Supplement: Supplementary file 1 [file clinpract-15-00169-s001.zip › clinpract-3826629-supplementary.pdf]

clinics and practice

MDPI

Review

Artificial Intelligence in Clinical Medicine: Challenges Across Diagnostic Imaging, Clinical Decision Support, Surgery, Pathology, and Drug Discovery

Eren Ogut

Department of Anatomy, Faculty of Medicine, Istanbul Medeniyet University, Istanbul 34700, Türkiye; erenogut@yahoo.com.tr or eren.ogut@medeniyet.edu.tr; Tel.: +0090-2162803333

Supplementary Table S1. PubMed Search Queries and Corresponding Hit Counts.

Domain	Search String (MeSH + Keywords)	Filters Applied	Records Retrieved
Diagnostic Imaging	("artificial intelligence"[MeSH] OR "machine learning" OR "deep learning" OR "neural network") AND ("radiology" OR "medical imaging" OR "CT" OR "MRI" OR "ultrasound" OR "X-ray")	Clinical trials, Human studies, Reviews	812
Clinical Decision Support	("artificial intelligence" OR "machine learning" OR "deep learning") AND ("clinical decision support" OR "CDSS" OR "electronic health records" OR "EHR" OR "risk prediction")	RCTs, Human studies, Reviews	456
Surgery	("artificial intelligence" OR "machine learning" OR "deep learning") AND ("surgery" OR "robotics" OR "intraoperative guidance" OR "surgical planning")	Clinical trials, Human studies	315
Pathology	("artificial intelligence" OR "machine learning" OR "deep learning") AND ("pathology" OR "histopathology" OR "digital pathology" OR "computational pathology")	Human studies, Reviews	229
Drug Discovery	("artificial intelligence" OR "machine learning" OR "deep learning") AND ("drug discovery" OR "drug development" OR "drug design" OR "virtual screening" OR "protein structure")	Reviews, Human studies	235
Total (before duplicates removed)	–	–	2,047

Supplementary Table S2. Categorization of Included Studies by Domain and Level of Evidence.

Domain	No. of Studies	Level I (RCTs)	Level II (Cohort)	Level III (Case-Control)	Level IV (Case Series)	Level V (Expert Opinion / Reviews)	Level VI (Mechanistic/Preclinical)
**Diagnostic Imaging**	80	12	25	18	15	10	0
**Clinical Decision Support**	20	4	6	4	3	3	0
**Surgery**	25	2	7	6	5	5	0
**Pathology**	20	3	5	4	5	3	0
**Drug Discovery**	25	0	5	3	7	10	0
**Total**	150	21	48	35	35	31	0

(Based on the Oxford Centre for Evidence-Based Medicine, 2011 – see: ***https://www.ncbi.nlm.nih.gov/books/NBK470182/***).

Disclaimer/Publisher's Note: The statements, opinions and data contained in all publications are solely those of the individual author(s) and contributor(s) and not of MDPI and/or the editor(s). MDPI and/or the editor(s) disclaim responsibility for any injury to people or property resulting from any ideas, methods, instructions or products referred to in the content.
